# Self-Assembly MXene-rGO/CoNi Film with Massive Continuous Heterointerfaces and Enhanced Magnetic Coupling for Superior Microwave Absorber

**DOI:** 10.1007/s40820-022-00811-x

**Published:** 2022-03-09

**Authors:** Xiao Li, Zhengchen Wu, Wenbin You, Liting Yang, Renchao Che

**Affiliations:** 1 Laboratory of Advanced Materials, Shanghai Key Lab of Molecular Catalysis and Innovative Materials, Fudan University, Shanghai, 200438 People’s Republic of China; 2 Department of Materials Science, Fudan University, Shanghai, 200438 People’s Republic of China

**Keywords:** MXene, Microwave absorption, Composite materials, Graphene, Electron holography

## Abstract

MXene, as a rising star of two-dimensional (2D) materials, has been widely applied in fields of microwave absorption and electromagnetic shielding to cope with the arrival of the 5G era. However, challenges arise due to the excessively high permittivity and the difficulty of surface modification of few-layered MXenes severely, which infect the microwave absorption performance. Herein, for the first time, a carefully designed and optimized electrostatic self-assembly strategy to fabricate magnetized MXene-rGO/CoNi film was reported. Inside the synthesized composite film, rGO nanosheets decorated with highly dispersed CoNi nanoparticles are interclacted into MXene layers, which effectively suppresses the originally self-restacked of MXene nanosheets, resulting in a reduction of high permittivity. In addition, owing to the strong magnetic coupling between the magnetic FeCo alloy nanoparticles on the rGO substrate, the entire MXene-rGO/CoNi film exhibits a strong magnetic loss capability. Moreover, the local dielectric polarized fields exist at the continuous hetero-interfaces between 2D MXene and rGO further improve the capacity of microwave loss. Hence, the synthesized composite film exhibits excellent microwave absorption property with a maximum reflection loss value of − 54.1 dB at 13.28 GHz. The electromagnetic synergy strategy is expected to guide future exploration of high-efficiency MXene-based microwave absorption materials.

**Supplementary Information:**

The online version contains supplementary material available at 10.1007/s40820-022-00811-x.

## Highlights

- The rGO/CoNi nanosheets embedded between the MXene layers can continue to serve as a conductive channel, ensuring carrier migration and proper conductive loss capability.
- Owing to the strong magnetic coupling between the magnetic FeCo alloy nanoparticles on the rGO substrate, the entire MXene-rGO/CoNi film exhibits a strong magnetic loss capability.
- Self-assembly MXene-rGO/CoNi films hold excellent microwave absorption performance − 54.1 dB at 13.28 GHz.

**Supplementary Information:**

The online version contains supplementary material available at 10.1007/s40820-022-00811-x.

## Graphical abstract

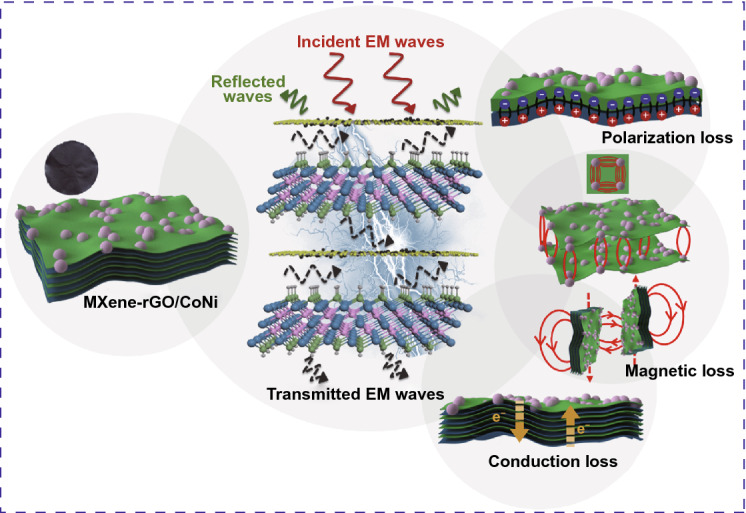

## Introduction

2D materials, such as hexagonal boron nitride [1], graphene [2], transition metal dichalcogenides (TMDC) [3], and MXene [4], have been extensively investigated as attractive and popular members in fields of microwave absorption (MA) [5, 6] and electromagnetic interference (EMI) shielding [7, 8] owing to their tunable active surface, outstanding electrical conductivity, and excellent mechanical strength. However, how to reasonably balance dielectric loss capacity with magnetic one is still a challenge, which hinders electromagnetic impedance optimization and absorption/shielding performance.

MXenes as a shining star of 2D materials have been widely reported in energy conversion and storage [9, 10], sensors [11, 12], gas separation [13, 14], water purification [15, 16], especially for MA [17] and EMI applications [18]. Due to the different etching conditions such as time, concentration, and temperature on their parent MAX phase, two typical MXene structures including few-layer and multi-layer can be obtained, respectively [4]. Few-layered MXenes have a 2D transparent nanosheet-like morphology similar to graphene and shows ultra-high conductivity. They are usually applied in the EMI field. In addition, few-layered Mxenes suffer from a serious self-stacking problem due to the attraction of Van der Waals forces [19]. Unlike graphene, it is difficult to directly modify the loading of exogenous components on the surface of the few-layered MXene. In comparison, multi-layered MXenes hold a unique accordion-like architecture, which provides the natural structural advantages for the further dissipation of incident electromagnetic waves. They are mostly used in the MA application. However, the MA performance of pure MXene is about − 11 dB and still needed to be enhanced [17]. Unlike EMI materials, MA materials need a moderate conductivity to meet the impedance matching conditions, so that more incident electromagnetic waves can enter into the material [20]. Therefore, the application of both few-layered and multi-layered MXene in the field of MA has obvious limitations. In addition, there are currently no effective approaches to combine the advantages of the few-layered and multi-layered MXene.

According to the microscopic absorption mechanism of MA materials, magnetic loss capability plays a vital role [21, 22]. The weak performance of pure MXene can be attributed to the fact that a single dielectric loss capability is not enough to completely dissipate electromagnetic waves. Feng et al. [23] prepared the composite of few-layered MXene and magnetic Ni nanoparticles by a simple hydrothermal method. The magnetic loss capability can be further controlled by changing the size of Ni nanoparticles. However, the narrow interlayer spacing of MXene (usually below 1.2 nm) hinders the intercalation of magnetic components. Importantly, graphene as another popular 2D material has been widely for MA due to its large number of active sites for decoration with magnetic components [24, 25]. Yu et al. fabricated the rGO/CoNi absorber by a hydrothermal method. Thanks to the effective combination between the alloy and the graphene substrate, the composite simultaneously exhibits excellent dielectric loss capability and magnetic loss capability. If it is possible to controllably insert graphene nanosheets modified with magnetic components between the layers of MXene nanosheets, and to prevent the self-stacking problems of 2D graphene and 2D MXene through alternating layered structures, it can be expected to obtain improved microwave absorption performance. Meanwhile, the order arrangement of few-layered MXene can be reshaped into a large-scale multi-layered MXene structure to give full play to their respective advantages.

Herein, a simple vacuum-assisted filtration is used to fabricate the magnetized MXene-rGO/CoNi film. The rGO/CoNi nanosheets embedded between the MXene layers can continue to serve as a conductive channel while expanding the interlayer spacing of MXene, ensuring carrier migration and proper conductive loss capability. The introduction of high-density magnetic CoNi nanoparticles greatly improves the magnetic loss capability of the MXene-rGO/CoNi film and enables it to have better impedance matching conditions. Due to the existence of the film substrate, the microscopic magnetic coupling behavior is successfully promoted from the nanometer size to the centimeter size, so that the MXene-rGO/CoNi film show the characteristics of being magnetized.

## Experimental and Calculation

### Materials

Ti_3_AlC_2_ MAX powders (≥ 98 wt% purity) were purchased from Jilin 11 Technology Co., Ltd. Cobalt chloride (CoCl_2_·6H_2_O), nickel chloride (NiCl_2_·6H_2_O), lithium fluoride (LiF), hydrazine hydrate (N_2_H_4_·H_2_O), ethanol (C_2_H_5_OH), ethylene glycol ((CH_2_OH)_2_), hydrochloric acid (HCl), sodium hydroxide (NaOH), hydrofluoric acid (HF) and diallyldimethylammonium chloride (PDDA) were purchased from Sinopharm Chemical Reagent Co., Ltd.

### Preparation of Few-Layered Ti_3_C_2_T_x_ MXene Solution

Firstly, 1.6 g of LiF was added to the mixed solution (15 mL HCl and 5 mL deionized water) and stirred to mix well. Secondly, 1 g of Ti_3_AlC_2_ MAX powder was continuously added to the above mixed solution and stirred at a constant temperature of 35 °C for 24 h. Finally, the above solution was diluted in 140 mL of deionized water and centrifuged to collect the upper layer solution [26].

### Preparation of rGO/CoNi Hybrids

Graphene oxide (GO) was prepared by a traditional Hummers method. rGO/CoNi composite was fabricated by a simple hydrothermal method [27]. Firstly, 50 mg of GO was sonicated in 100 mL ethylene glycol solution to obtain few-layered nanosheets. Secondly, 1 mmol of CoCl_2_·6H_2_O and 1 mmol of NiCl_2_·6H_2_O were added in the above solution and sonicated for 4 h. Thirdly, 25 mL of N_2_H_4_·H_2_O and 25 mmol of NaOH were added in the above solution and stirred to mix well. Finally, the solution was stirred at a constant temperature of 120 °C for 1 h. The black precipitate was collected by a magnet and wash it several times with deionized water and ethanol [28].

### Preparation of Flexible MXene-rGO/CoNi Films

MXene-rGO/CoNi films was prepared by electrostatic self-assembly and vacuum-assisted filtration method. Firstly, rGO/CoNi-PDDA solution was added drop by drop to 60 mL of few-layered MXene solution. Secondly, the above mixed solution is placed into a vacuum filtration device to prepare MXene-rGO/CoNi film (denoted as MXene-xrGO/CoNi, where x is the mass ratio of rGO/CoNi in the mixed solution).

### Characterization

The crystal structure information of all prepared samples was measured by an x-ray powder diffraction (XRD) with Ni-filtered Cu Kα radiation (40 kV, 40 mA). The morphology information was analyzed by scanning electron microscopy (SEM) with a Hitachi S-4800 field-emission scanning electron microscope, transmission electron microscopy (TEM), high resolution TEM (HRTEM), selected-area electron diffraction (SAED), and off-axis electron holography with a JEM-2100F transmission electron microscope. The electromagnetic parameters information was characterized by a coaxial ring method with a N5230C vector network analyzer. The film was cut into dozens of coaxial pieces with external and inner diameters of 3.0 and 7.0 mm, respectively. These rings were stacked within a mold, followed by adding the molten paraffin (10 wt%). Finally, the mold was vigorously pressed to obtain the test ring (Fig. S1). The microwave absorption performance was calculated by the following formula [29, 30].

1 $$ Z = \left| {Z_{{{\text{in}}}} /Z_{0} } \right| = \sqrt {\mu_{{\text{r}}} /\varepsilon_{{\text{r}}} } \tanh [j(2\pi fd/c)\sqrt {\varepsilon_{{\text{r}}} \mu_{{\text{r}}} } ] $$

2 $$ {\text{RL}}\left( {{\text{d}}B} \right) = 20\lg \left| {(Z_{{{\text{in}}}} - Z_{0} )/\left( {Z_{{{\text{in}}}} + Z_{0} } \right)} \right| $$

where *Z*_0_ is the impedance of free space, Zin is the input impedance, *f* is the frequency of electromagnetic wave, *d* is the thickness of the absorbing layers and *c* is the light velocity in free space.

## Results and Discussion

**Fig. 1:**
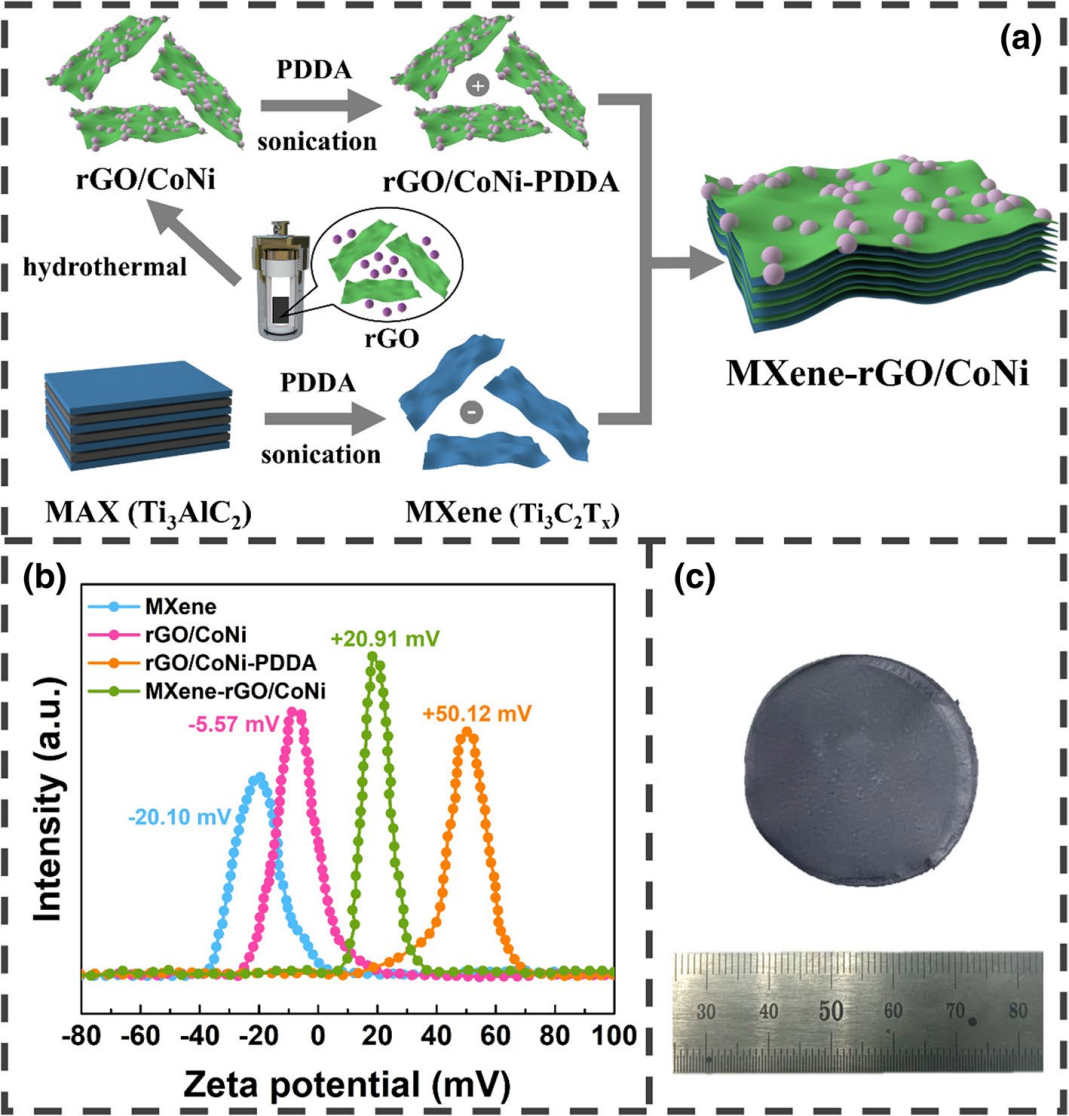
**a** Schematic illustration for the synthesis of the MXene-rGO/CoNi hybrids; **b** zeta potential of MXene, rGO/CoNi, rGO/CoNi-PDDA and self-assembled MXene-rGO/CoNi hybrid; **c** photograph of the MXene-rGO/CoNi film

Figure 1a describes the preparation of the magnetized MXene-rGO/CoNi films. Firstly, rGO/CoNi powder is fabricated by the simple hydrothermal method and can be easily attracted by magnets, indicating its intrinsic magnetism (Fig. S2). Due to the presence of oxygen-containing functional groups, GO/CoNi nanosheet is negatively charged (− 5.57 mV). For subsequent electrostatic self-assembly [31], the GO/CoNi nanosheet is immersed in the 0.01 wt% PDDA solution and sonicated to make it sufficiently stable. Thus, the rGO/CoNi-PDDA is positively charged with a zeta potential of + 50.12 mV (Fig. 1b). Due to the existence of surface functional groups produced during the intense etching process, the zeta potential of few-layered MXene is measured to be − 20.10 mV (Fig. 1b) [31]. Finally, the flexible film is prepared by the simple vacuum-assisted filtration method (Fig. 1c).

**Fig. 2:**
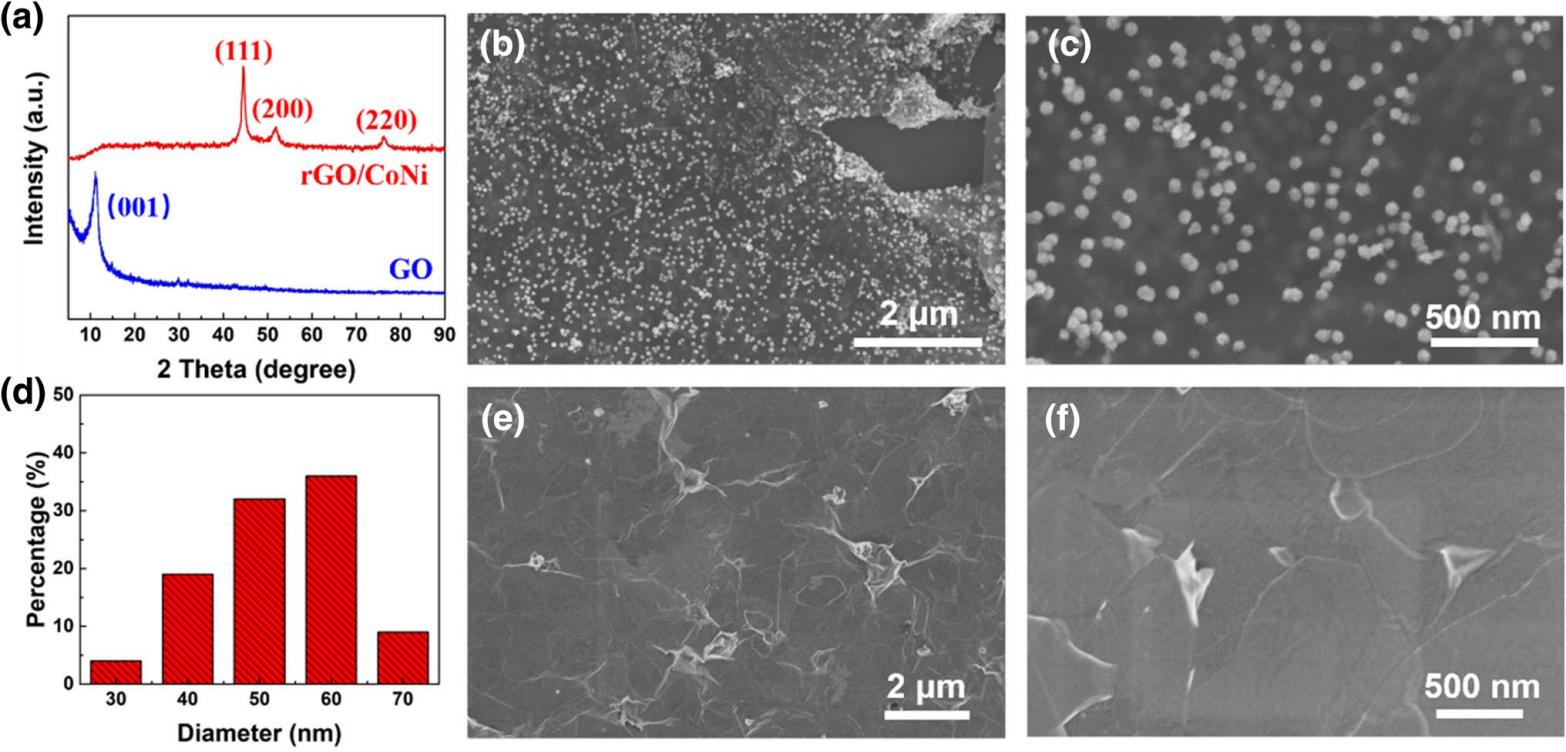
**a** XRD patterns of GO and rGO/CoNi; **b**, **c** SEM images of rGO/CoNi; **d** size distribution of CoNi in rGO; **e**, **f** SEM images of MXene

**Fig. 3:**
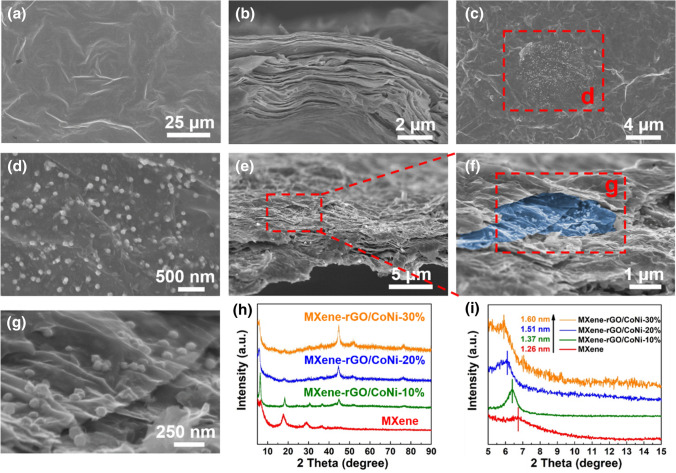
**a** SEM image and **b** cross-sectional SEM image of pure MXene film; **c**, **d** SEM images and **e**–**g** cross-sectional SEM images of MXene-rGO/CoNi film; **h** XRD patterns of MXene, MXene-10%rGO/CoNi, MXene-20%rGO/CoNi and MXene-30%rGO/CoNi; **i** magnification of XRD patterns in **h**

The crystallographic structure of GO and rGO/CoNi are characterized by XRD technique (Fig. 2a). A sharp diffraction peak at 11° is observed in GO sample, corresponding to the (001) plane with an interplanar distance of 0.85 nm [28]. For rGO/CoNi composite, three diffraction peaks at 44.5°, 51.7°, and 76.2° can be well related to (111), (200), and (220) planes, which is consistent with the face center cubic structure of Co (JCPSD No. 15-0806) and Ni (JCPDS No. 04-0850) [32], respectively. After in-situ nucleation in hydrothermal reaction, CoNi nanoparticles with a size of ~ 50 nm are anchored uniformly on the rGO nanosheets (Fig. 2b–d). Since the precursor is fully ultrasonically dispersed when it is loaded on the graphene substrate, the prepared magnetic CoNi alloy has good dispersion uniformity and no obvious magnetic agglomeration. The SEM images of raw MAX and etched few-layered MXene are presented in Figs. S3 and 2e, f, suggesting the successful lamination process. Besides, the AFM figure of delaminated MXene shows its thickness is 2.1 nm, demonstrating its few-layered structural feature (Fig. S4). Figures 3a, b and S5 show the SEM images of pure MXene and pure GO film, respectively. In the top-view images, some wrinkled areas are observed on the surface of pure MXene and GO films, which is caused by the filtration process. In the cross-sectional images, both pure MXene and GO films show a similar well-arranged layered architecture. After the intercalation of rGO/CoNi nanosheets, the MXene-rGO/CoNi films still maintain a similar layered structure, as shown in Fig. 3e–g. From the top-view images of MXene-rGO/CoNi films, the cracked MXene nanosheets are decorated with rGO/CoNi hybrids that prove the successful introduction (Figs. 3c, d and S6). The CoNi nanoparticles on the rGO substrate still keep the uniformly high distribution without obvious agglomeration (Fig. 3d). Cross-sectional SEM images of MXene-rGO/CoNi films indicate the alternative stacking of 2D MXene and 2D rGO/CoNi, indicating the successful preparation of self-assembly structure. The rGO/CoNi nanosheet is successfully inserted between the adjacent layers of MXene nanosheets. Both mitigative restacking issue and increased exposed surface area could be obtained simultaneously. The film demonstrates the obvious magnetism. When the mass percentage of rGO/CoNi is 30 wt%, the saturation magnetization is 27.0 emu g^−1^, much lower than that of pure CoNi, due to the existence of nonmagnetic component (Fig. S7). XRD is measured for pure MXene and different MXene rGO/CoNi films to analyze the interlayer spacing change. The obvious diffraction peak at 6.6° of (002) plane is observed in the pure MXene film, corresponding to an interlayer spacing of 1.26 nm (Fig. 3h). Increasing the mass percentage of rGO/CoNi from 0 to 30 wt%, the position of the (002) diffraction peak shifts from 6.6° to 5.8° in the product. Accordingly, the corresponding interlayer spacing is increased from 1.26 to 1.60 nm. For pure MXene film, neighboring nanosheets are closely stacked due to the van der Waals forces to form a neatly arranged multi-layered morphology. After the intercalation of rGO/CoNi nanosheets, the adjacent layer structure of MXene is destroyed while maintaining the original multi-layered architecture. Therefore, the serious restacking issue of MXene nanosheets could be effectively avoided. By increasing the number of rGO/CoNi introduced, more MXene nanosheets are separated that forming continuous large-scale hetero-interfaces in the whole.

**Fig. 4:**
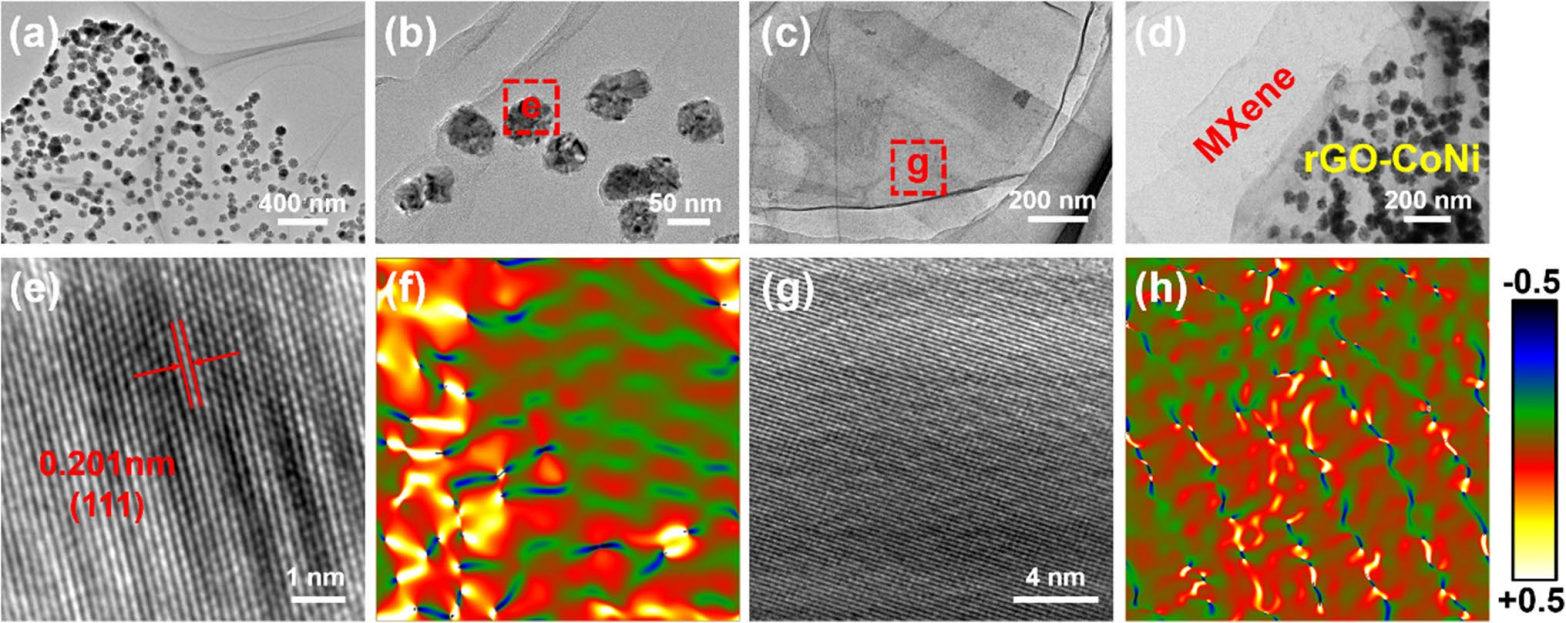
TEM images of **a**, **b** rGO/CoNi, **c** MXene and **d** MXene- rGO/CoNi; HRTEM images of **e** CoNi in the rGO and **f** corresponding strain maps; HRTEM images of **g** MXene and **h** corresponding strain maps

As shown in Figs. 4a, b and S8a, the rGO nanosheets have a transparent morphology. Many wrinkled areas can be observed at the edges. A large number of spherical CoNi nanoparticles with a diameter of about 50 nm are tightly attached to the rGO substrate, which is in good agreement with the SEM result. Adjacent CoNi nanoparticles are separated from each other without severe agglomeration. After a long time of ultrasonic and stirring treatment, the CoNi nanoparticles still do not fall off or self-agglomeration. These results confirm the strong interaction between the in-situ hydrothermally grown CoNi nanoparticles and rGO substrate. The SAED pattern of rGO/CoNi composites shows a face-centered cubic crystal structure and high crystalline feature, which is in good agreement with the XRD result (Fig. S8b) [32]. The interplanar distance of CoNi nanoparticles is 0.201 nm, corresponding to the (111) plane (Fig. 4e). After strongly HF etching, the monolayer MXene nanosheet is similar to the rGO nanosheets with the large and transparent morphology (Fig. 4c). For MXene-rGO/CoNi composites, MXene nanosheets and rGO/CoNi flakes are closely attached to each other due to the electrostatic interactions (Fig. 4d). Compared with the CoNi nanoparticles that decorated in the rGO substrate, the free-growing CoNi nanoparticles have increased in size by about 7 times without the confinement effect of rGO (Fig. S9a). In addition, the CoNi alloys grown in free space can attract each other due to their intrinsic magnetic characteristics and cause serious agglomeration, thereby losing a large amount of magnetically active surface. Moreover, massive defects form in CoNi during the nucleation process, which can be corresponded to the stain fields diagram (Fig. 4f) [33, 34]. Similarly, numerous points with reversal color are detected in Fig. 4h, indicating the typical dislocations due to the presence of surface functional groups (–OH/–F) in the defect-rich MXene nanosheets. The incident electromagnetic wave passing through these active defects could cause a strong dipole polarization behavior, which helps to improve its dielectric loss capability.

**Fig. 5:**
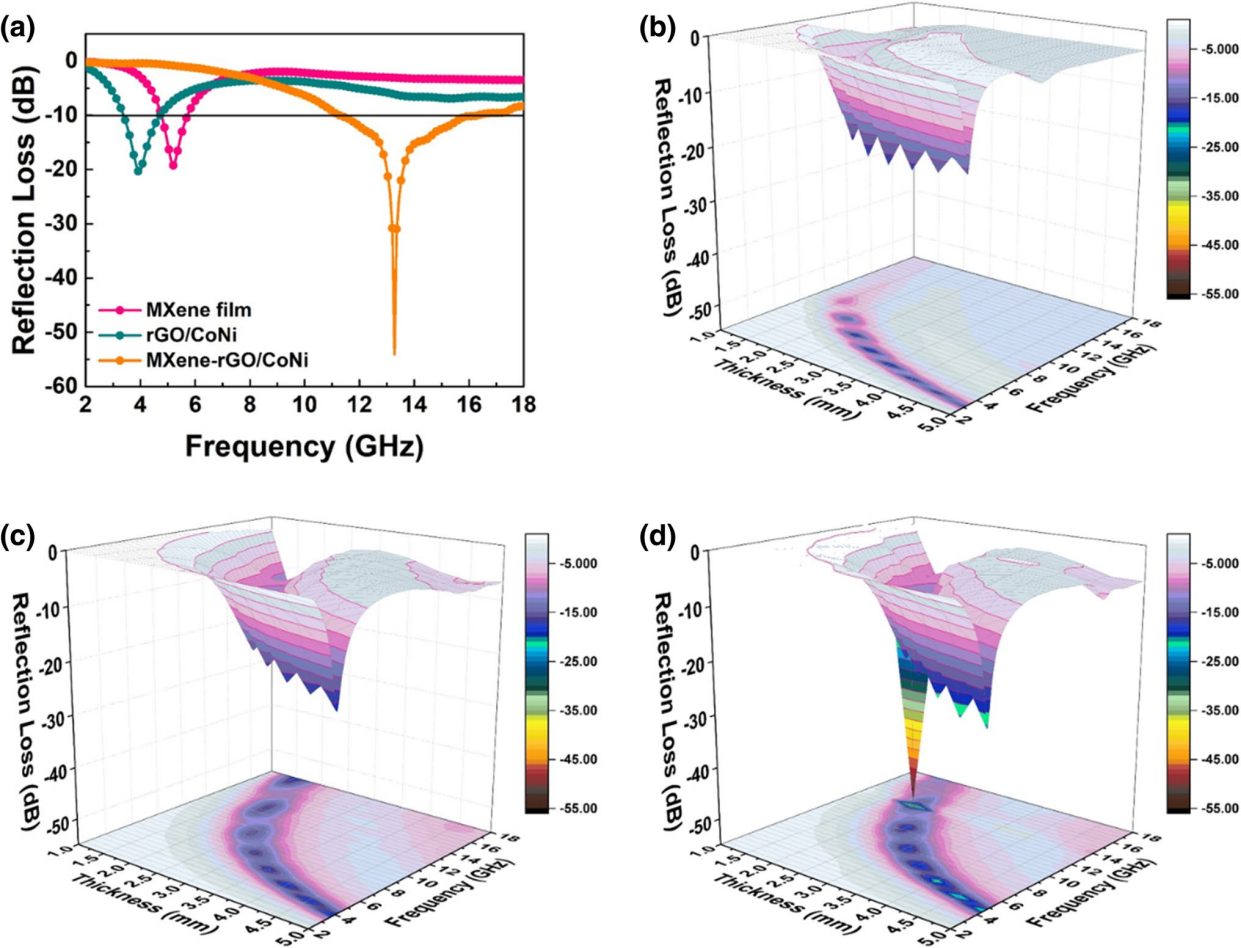
**a** Three samples with their respective highest RL values; 3D plots of RL of **b** MXene, **c** rGO/CoNi, **d** MXene-rGO/CoNi films

Figure 5a shows the reflection loss (RL) curves of MXene, rGO/CoNi and MXene-rGO/CoNi films with their respective highest MA property in 2–18 GHz. Notably, the highest RL value of MXene-rGO/CoNi film holds − 54.1 dB at 13.28 GHz, those of each component and some MXene-based absorbents reported previously (Table S1). In addition, Fig. 5b–d shows the 3D presentations of calculated theoretical RL values of the as-prepared composites with various thicknesses (1–5 mm). As the thickness increases, the corresponding strongest absorption peaks of the three samples all shift to the low-frequency range.

**Fig. 6:**
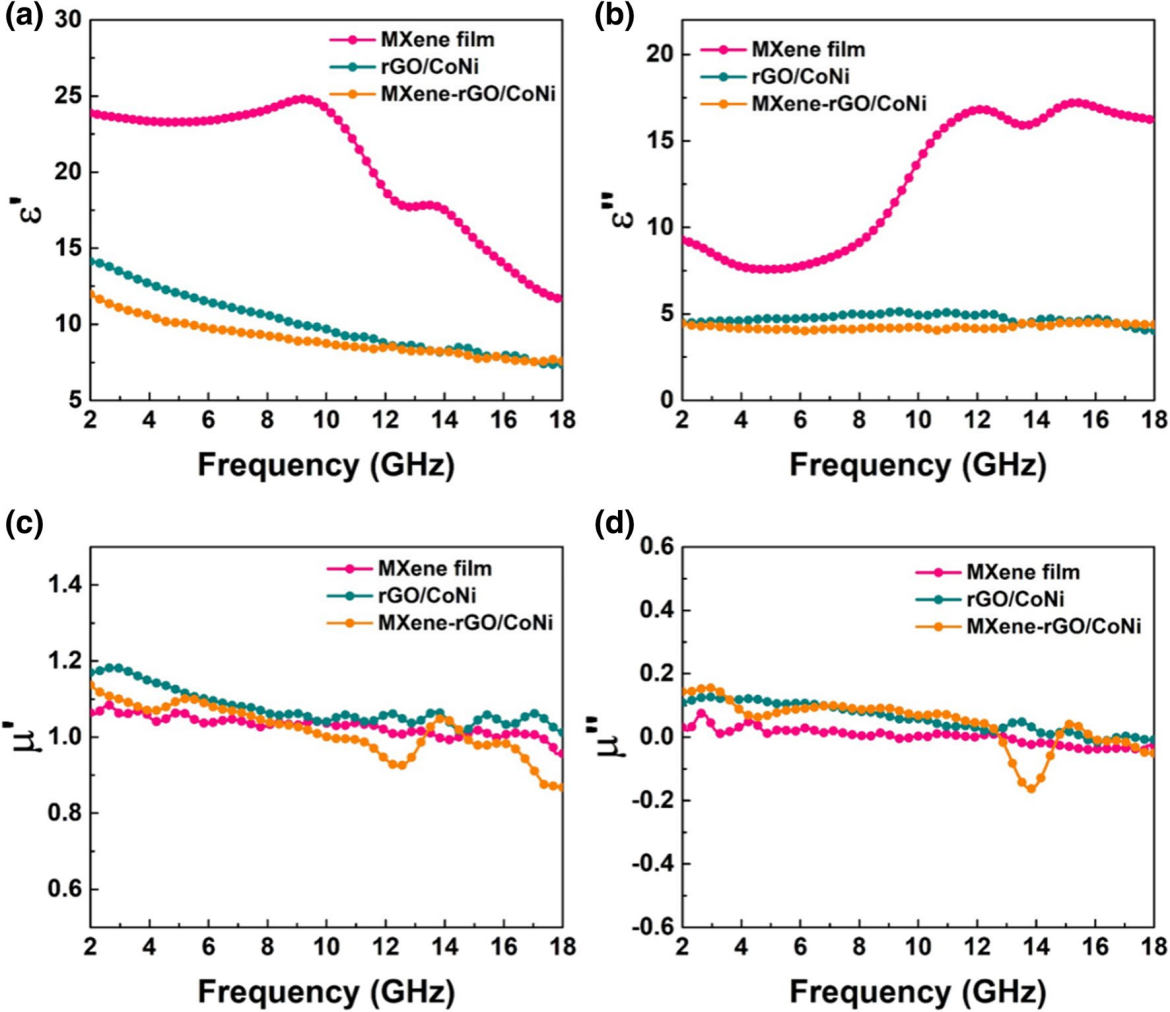
**a** real part and **b** imaginary part of permittivity vs frequency, **c** real part and **d** imaginary part of permeability vs frequency of MXene, rGO/CoNi and MXene-rGO/CoNi films

The MA performance is directly determined by the complex permittivity ($\varepsilon_{r} = \varepsilon ^{{\prime}{}} - j\varepsilon ^{{\prime\prime}{}}$) and permittivity ($\mu_{r} = \mu ^{{\prime}{}} - j\mu ^{{\prime\prime}{}}$). In principle, $\varepsilon ^{\prime}$ represents the internal polarization behavior of the material [35], while $\varepsilon ^{\prime\prime}$ depends on the material’s electrical conductivity [17]. As expected, pure MXene film composed of few-layered MXene has the highest complex permittivity due to its intrinsic ultra-high electrical conductivity. $\varepsilon ^{\prime}$ and $\varepsilon ^{\prime\prime}$ values range from 25 to 15 and 10 to 15, respectively (Fig. 6a–b). Two obvious peaks in the $\varepsilon ^{\prime}$ curve at 9 GHz and 14 GHz can be derived from the interface polarization behavior caused by the well-arranged layered structure. However, excessive dielectric loss capability and insufficient magnetic loss capability directly led to serious impedance matching imbalance, making massive surface reflection of electromagnetic waves. This is the main reason for the weak MA performance of pure MXene film. After introducing rGO/CoNi nanosheets into the interlayer of pure MXene film, both $\varepsilon ^{\prime}$ and $\varepsilon ^{\prime\prime}$ values are significantly decreased due to the reduced conductivity in MXene-rGO/CoNi film. rGO has a transparent 2D nanosheets structure similar to few-layered MXene. When rGO is intercalated between the layers of pure MXene film, the appearance and morphology still maintain the original well-arranged structure. However, the micro-continuous layered structure of pure MXene is destroyed due to the difference in the crystal structure. It hinders the migration of electrons between adjacent layers, resulting in a significant decrease in the conductivity of MXene-rGO/CoNi film. Conversely, the embedding of MXene between rGO layers also destroys the continuity of the rGO layered structure in turn. Therefore, both the $\varepsilon ^{\prime}$ and $\varepsilon ^{\prime\prime}$ values of rGO/CoNi are higher than those of MXene-rGO/CoNi film. For the complex permeability value, the $\mu ^{\prime}$ and $\mu ^{\prime\prime}$ values of pure MXene film are close to 1 and 0 due to the absence of magnetic component (Fig. 6d, e). Comparatively, both $\mu ^{\prime}$ and $\mu ^{\prime\prime}$ values of other two CoNi-based films are significantly improved due to the magnetic property. The magnetic loss mechanisms in microwave frequency include resonance and current eddy. The C0 value, which equals to $\mu ^{\prime\prime}\left( {\mu ^{\prime}} \right)^{2} f^{ - 1}$, has been employed to reveal the origin of magnetic loss (Fig. S10). Generally, the eddy current occurs within the frequency bandwidth where the C0 is constant, otherwise, the resonance plays the leading role. For both the magnetic samples, their C0 values decrease with the increase in frequency and remain almost unchanged at the frequency beyond 16.0 GHz. Therefore, resonance losses in both natural and exchange types dominate their magnetic response process.

**Fig. 7:**
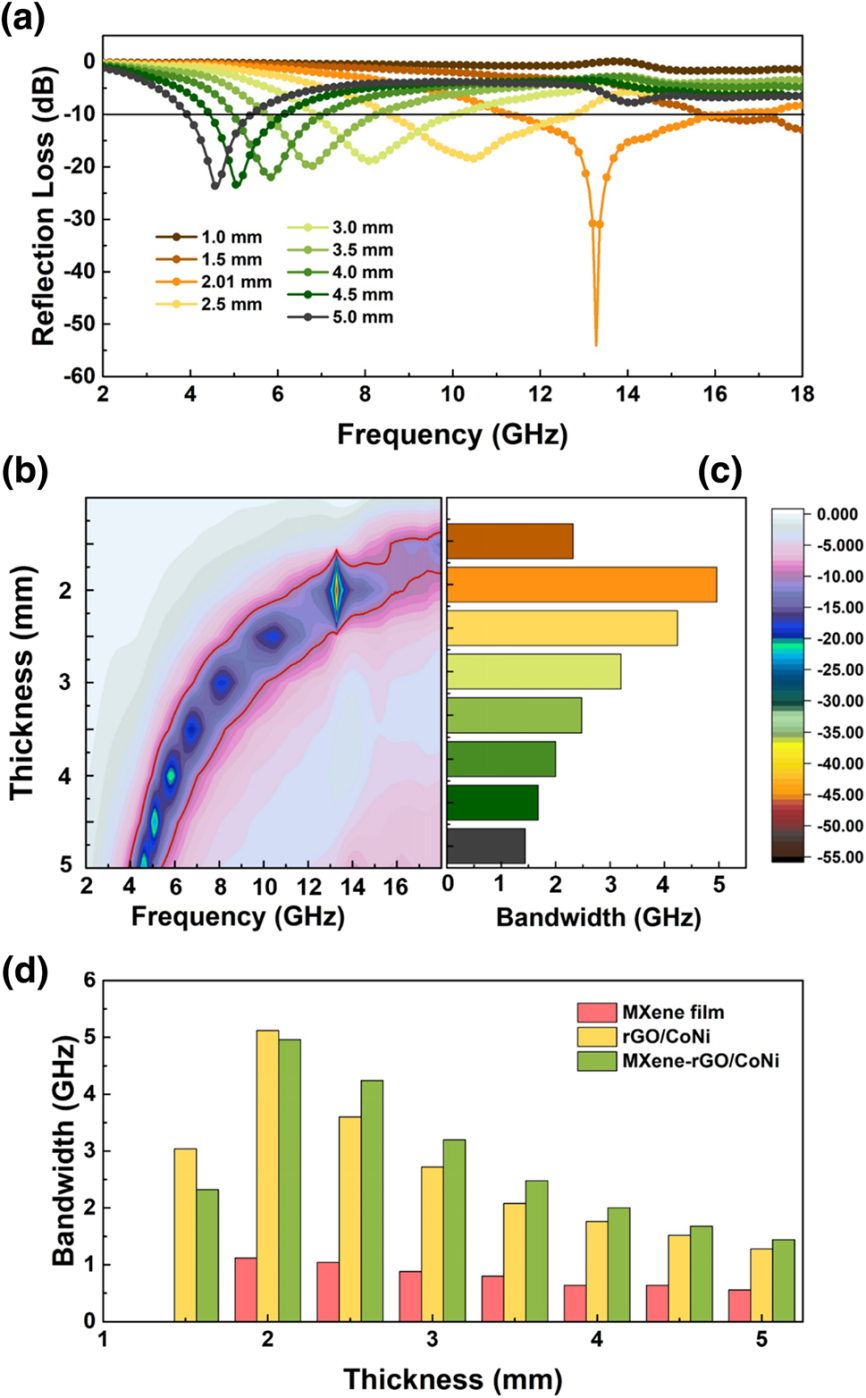
**a** RL curves vs frequency, **b** 2D representation of RL values and **c** bandwidth values of MXene-rGO/CoNi films, **d** compared bandwidth of MXene, rGO/CoNi and MXene-rGO/CoNi films

Taking the needs of practical applications into account, the value of effective absorption bandwidth should also be paid attention to while focusing on the strong RL values (Fig. 7). With a thickness of 2.1 mm, MXene-rGO/CoNi film has both the strongest reflection loss and the widest effective absorption bandwidth. Moreover, at the high frequency from 8.3 to 18.0 GHz, the MXene-rGO/CoNi demonstrates wider effective absorption bandwidth. Therefore, the main performance advances of our material design include the enhancement of absorption intensity and the improvement of high-frequency absorption.

**Scheme 1:**
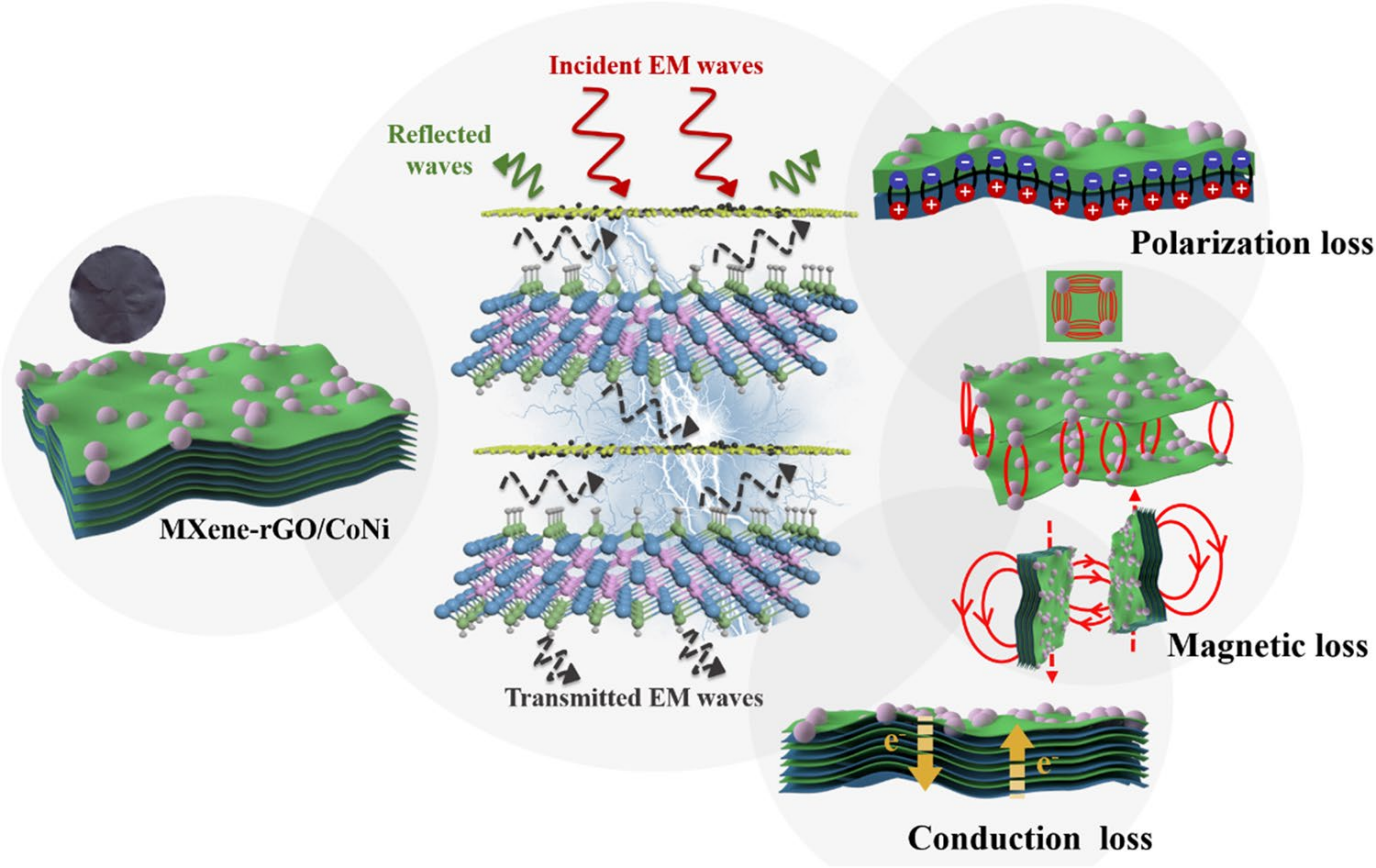
Schematic illustration of MA mechanisms for MXene- rGO/CoNi films

The microscopic absorption mechanism of self-assembly magnetized MXene-rGO/CoNi film can be summarized as the following three points (Scheme 1).

### Suitable Conduction Loss Capability Caused by the Continuous 2D/2D Interfaces

Few-layered MXene has a large and thin 2D structure, which is difficult to surface modification. While the special accordion-like morphology of multilayered structures can effectively extend the dissipation path of incident electromagnetic waves. Re-stacking the few-layered MXene into an ordered layered structure by means of vacuum-assisted filtration can fully combine their advantages. However, the well-arranged MXene-rGO/CoNi film composed of few-layered MXenes still has good conductivity, which directly leads to the imbalance of the overall impedance matching and the inability of electromagnetic waves to enter the material. On one hand, the embedding of 2D rGO can effectively reduce the excessively high dielectric loss capability of pure MXene material. On the other hand, it still provides a conductive path for the migration of carriers.

### Strong Polarization Loss Capability Provided by the Massive Hetero-Interfaces

**Fig. 8:**
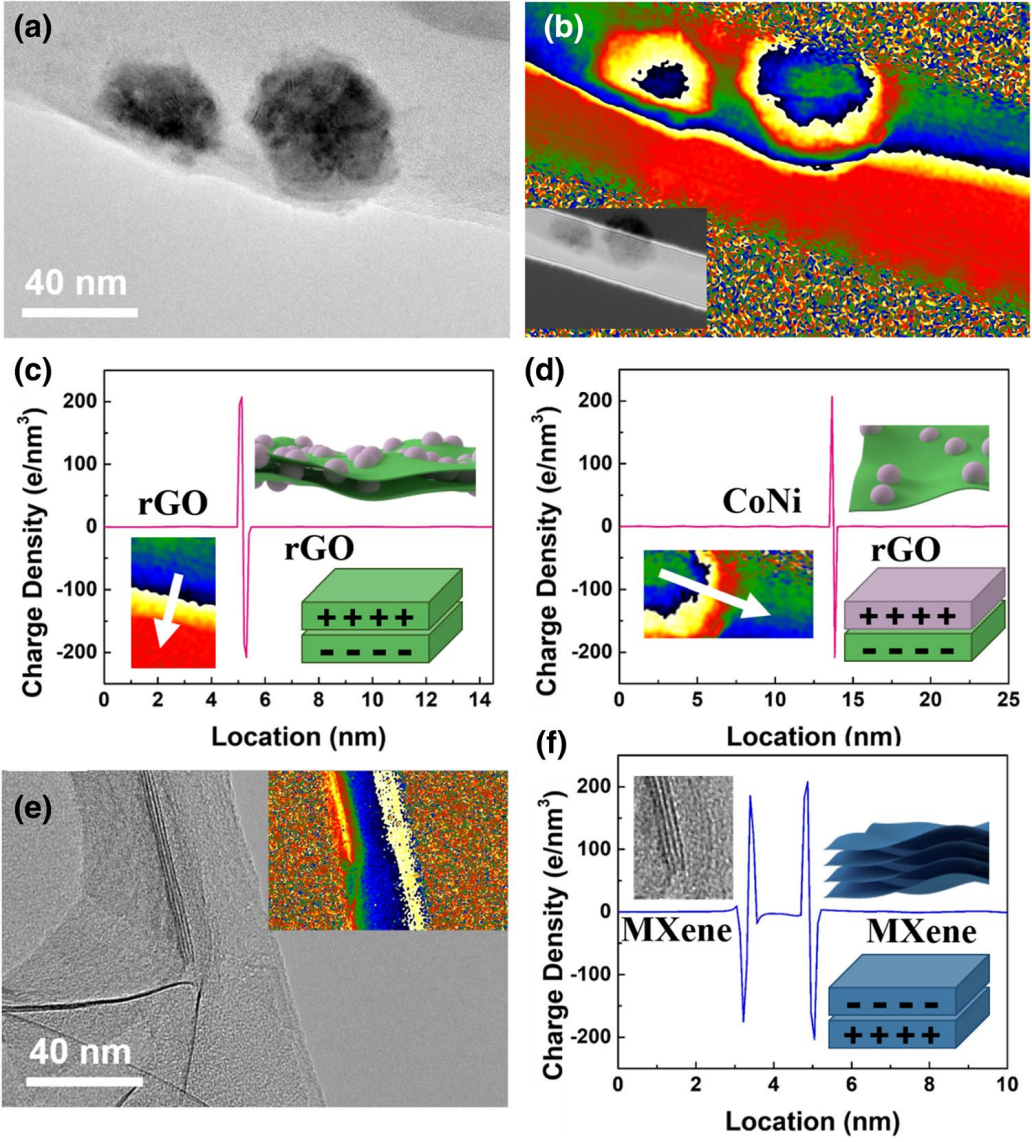
**a** TEM image, **b** charge density images and **c**, **d** charge density profiles in the region of the white arrow of rGO/CoNi; **e** TEM image and **f** charge density profiles in the region of the white arrow of MXene

In order to fully analyze the difference in dielectric loss capability of MXene, rGO/CoNi and MXene-rGO/CoNi film, heterogeneous interfaces are discussed in detail (Fig. 8). After the in-situ hydrothermal nucleation reaction, the magnetic CoNi nanoparticles with high density and good dispersibility grow tightly on the rGO substrates. Each position where the CoNi nanoparticles is in contact with the rGO nanosheets is considered to be a micro-capacitor model. The electromagnetic wave can be quickly dissipated in the form of heat energy when passing through the plane layer. In order to verify this concept, the advanced electron holography method is applied to visually describe it [36]. The charge density distribution can be obtained from $\rho \left( \chi \right) = - \varepsilon_{\gamma } \varepsilon_{0} \frac{{\partial^{2} \nu \left( \chi \right)}}{{\partial \chi^{2} }}$. Figure 8b is the reconstructed charge density distribution diagram corresponding to Fig. 8a. The multiple colors indicate the different degrees of charge density accumulation in this area. On the vertical plane, two distinct peaks can be observed at the junction of adjacent rGO nanosheets. Therefore, a strong interface polarization behavior is formed at the rGO-rGO interface as previously envisaged. A similar phenomenon can also be observed between neighboring MXene nanosheets on the vertical plane. In addition, the CoNi nanoparticles and rGO substrate on the horizontal plane also have a large amount of carrier accumulation behavior, Therefore, the intensive polarization induced by the large number of interfaces in MXene-rGO/CoNi film leads to excellent microwave absorption performance.

### Enhanced Magnetic Loss Capability Contributed by Highly Dispersed Magnetic CoNi Nanoparticles

**Fig. 9:**
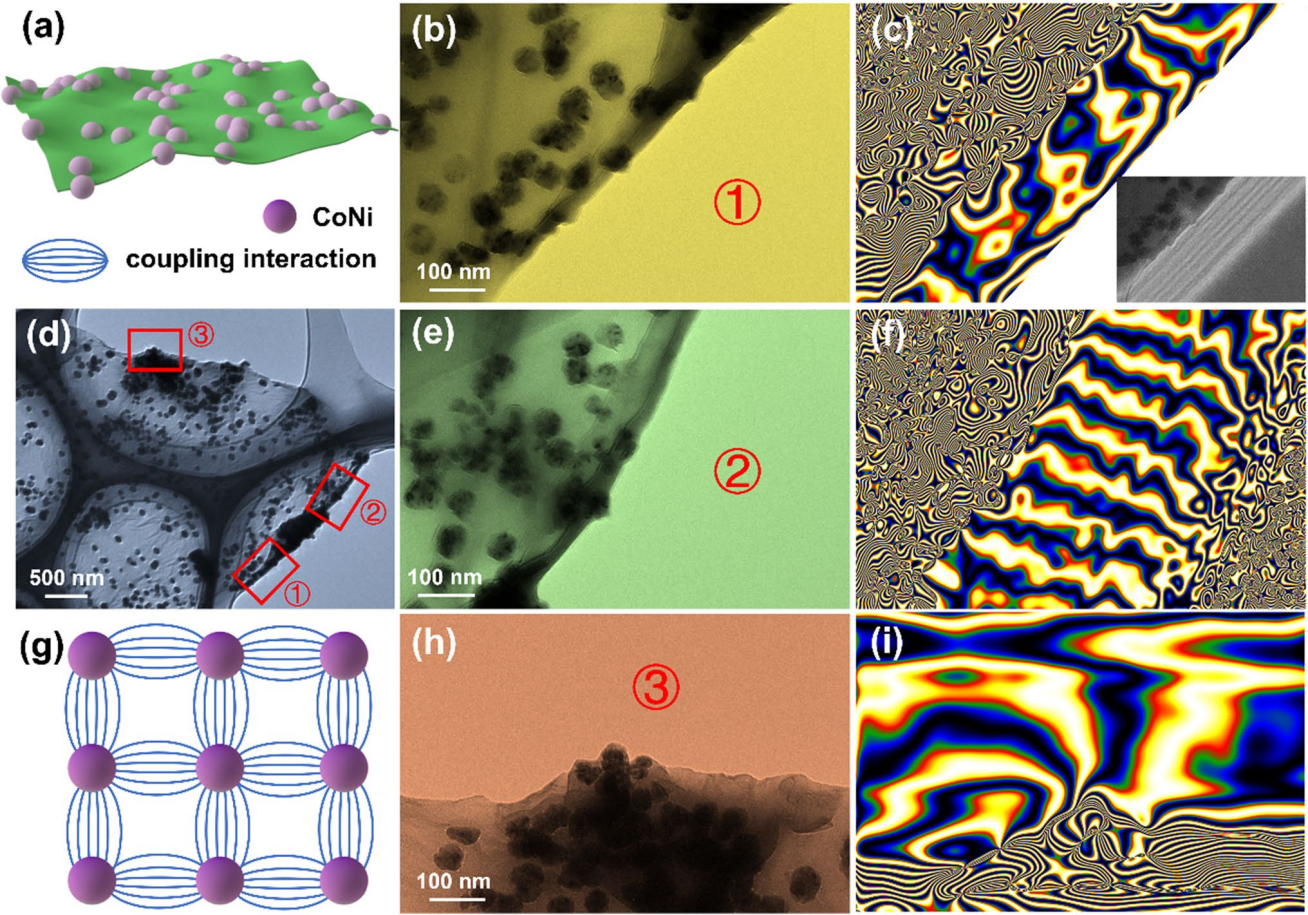
**a**, **g** Schematic illustration, **b**, **d**, **e**, **h** TEM images and **c**, **f**, **i** magnetic flux lines of rGO/CoNi sample

In particular, magnetic loss capability plays another important role in the microscopic absorption mechanism [37]. Massive highly dispersed CoNi nanoparticles are firmly embedded on a large and thin rGO substrate, so that the entire MXene-rGO/CoNi film shows the characteristics of being magnetized (Fig. 9). On the horizontal plane, the CoNi nanoparticles emit many obvious magnetic flux lines to the free space, which proves its significantly improved magnetic loss capability. Part of the adjacent magnetic flux lines fuses to form a semicircular structure, as shown in Fig. 9i, indicating the existence of magnetic coupling behavior. The difference from the traditional electro-magnetic composite method is that the magnetic coupling behavior existing on the film can be expanded from the original nanometer level to the centimeter level.

## Conclusions

In summary, the use of vacuum-assisted filtration method to restack a few-layered MXene into an orderly layered structure greatly extends the dissipation path of incident electromagnetic waves. rGO with a similar structure is selected as the intercalation modifier, which destroys the continuity of the original pure MXene film and reduces the excessively high conductivity to make it more in line with the design requirements of microwave absorption materials. By changing the amount of intercalation of rGO, precise control of its electromagnetic parameters can be achieved. In addition, a large number of highly dispersed magnetic alloy particles are firmly attached to the surface of the graphene so that the entire film exhibits magnetic characteristics and greatly improves the magnetic loss capability. The magnetic coupling behavior is increased from the original nanometer scale to the centimeter scale, so that the MXene-rGO/CoNi film exhibit the distinct microwave absorption property with strong RL values and wide EAB values simultaneously.

## Supplementary Information

Below is the link to the electronic supplementary material.

Supplementary file1 (PDF 758 KB)
