# Supplementary material for: Self-Assembly MXene-rGO/CoNi Film with Massive Continuous Heterointerfaces and Enhanced Magnetic Coupling for Superior Microwave Absorber
Source: Nanomicro Lett. 2022 Mar 9;14:73. doi: 10.1007/s40820-022-00811-x (PMC8907377; doi:10.1007/s40820-022-00811-x)
Supplement: Supplementary file 1 — Supplementary file1 (PDF 758 KB) [file 40820_2022_811_MOESM1_ESM.pdf]

Supporting Information for

Self-Assembly MXene-rGO/CoNi Film with Massive Continuous Heterointerfaces and Enhanced Magnetic Coupling for Superior Microwave Absorber

Xiao Li[1,#], Zhengchen Wu[1,#], Wenbin You[1], Liting Yang[1], and Renchao Che[1,2,*]

[1] Laboratory of Advanced Materials, Shanghai Key Lab of Molecular Catalysis and Innovative Materials, Fudan University, Shanghai 200438, P. R. China

[2] Department of Materials Science, Fudan University, Shanghai 200438, P. R. China

[#]Xiao Li and Zhengchen Wu contributed equally to this work

*Corresponding author. E-mail: rcche@fudan.edu.cn (Renchao Che)

Supplementary Figures

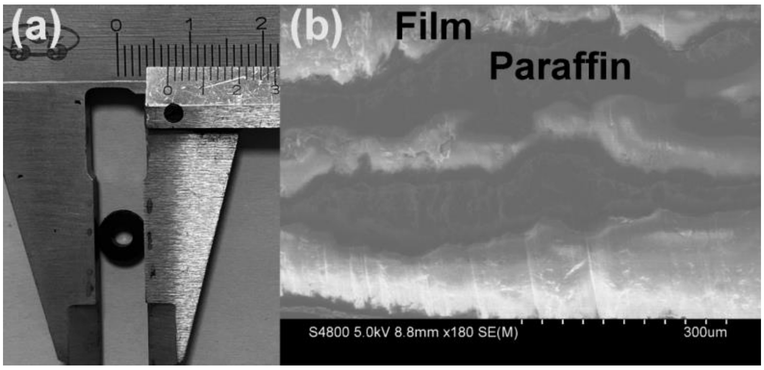

Fig. S1 (**a**) The digital photo and (**b**) cross-section SEM image of ring sample for the measurement of EM parameters

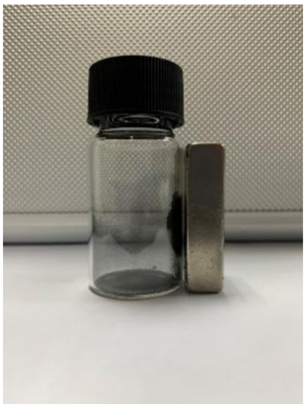

Fig. S2 Image of rGO/CoNi powder

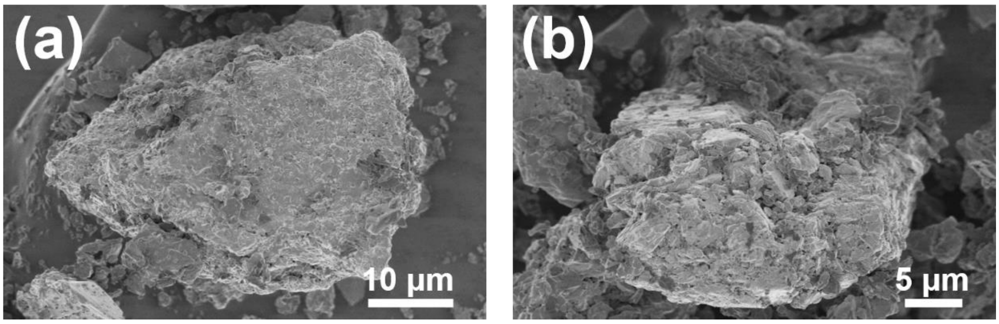

Fig. S3 SEM image of MAX

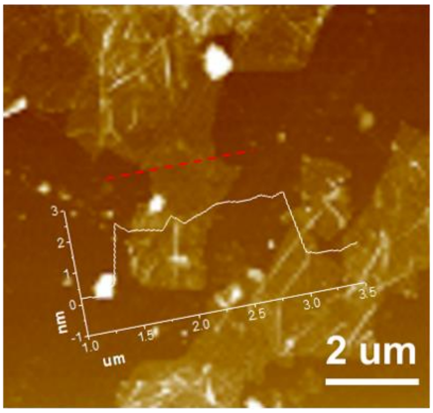

Fig. S4 The AFM image of $Ti_3C_2T_x$ MXene

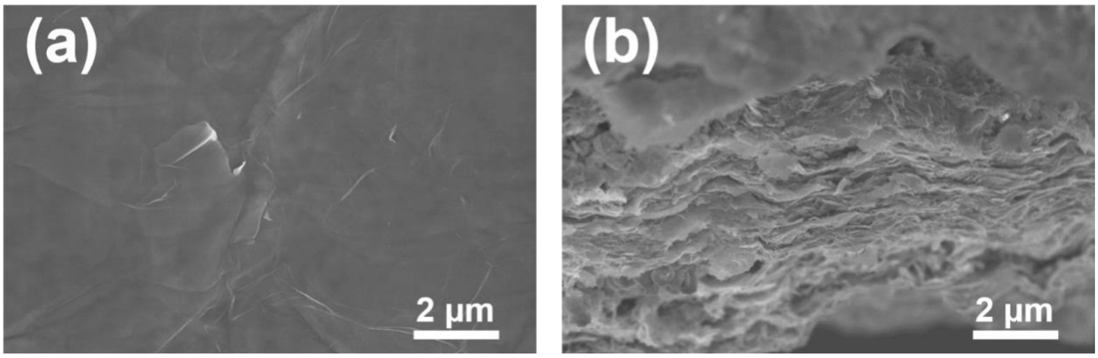

Fig. S5 (**a**) SEM image and (**b**) cross-sectional SEM image of pure GO film

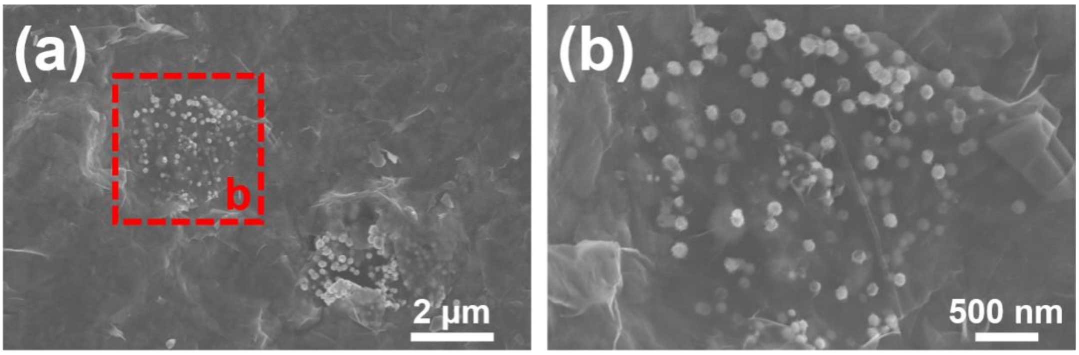

Fig. S6 (**a**) SEM images and (**b**) magnified region of red line in (**a**) of of MXene-rGO/CoNi film

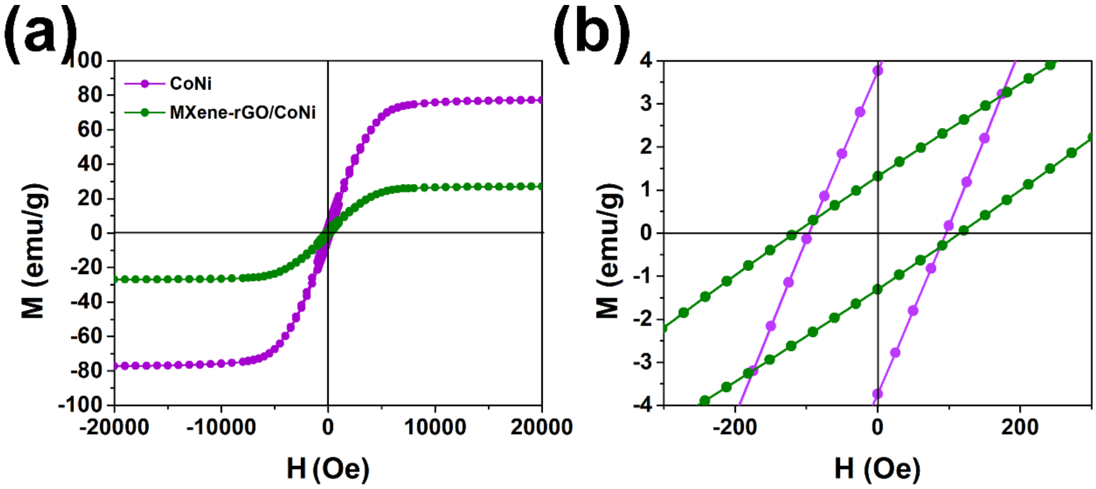

Fig. S7 The hysteresis loop of CoNi and MXene-rGO/CoNi

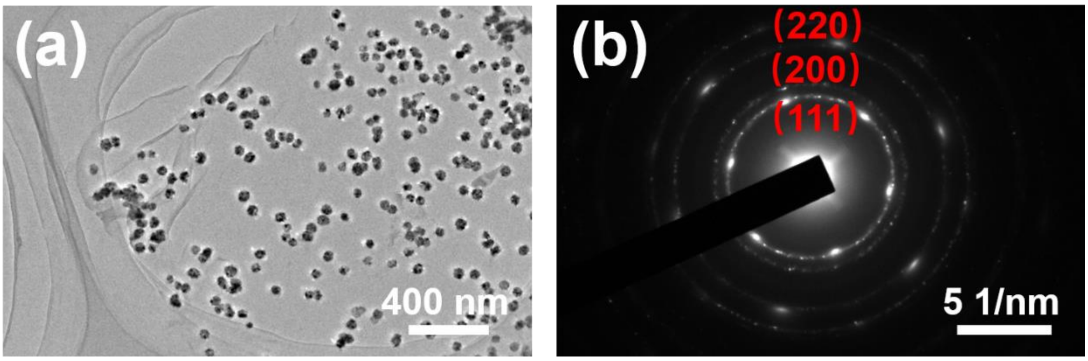

Fig. S8 (**a**) TEM image and (**b**) SAED pattern of rGO/CoNi powder

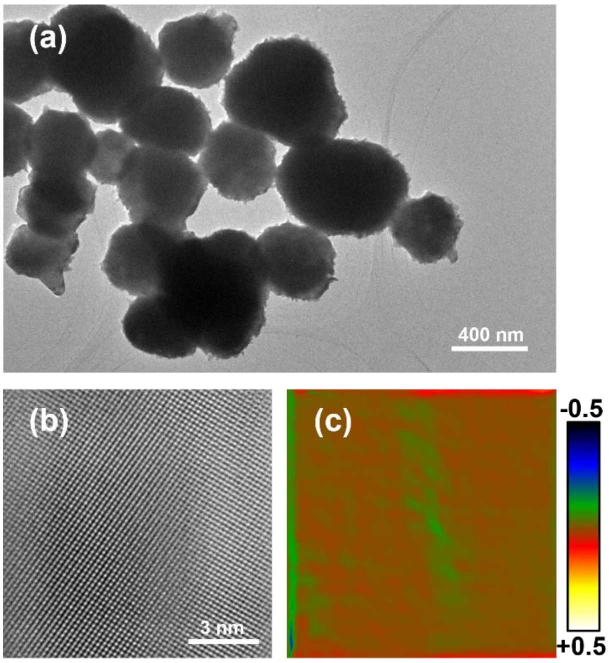

Fig. S9 (**a**) TEM image, (**b**) HRTEM and (**c**) corresponding strain maps of free-growing CoNi without rGO

Table S1 The maximum reflection loss (RL_{max}) and effective absorption bandwidth (EAB) of MXene-rGO/CoNi and some $Ti_3C_2T_x$ MXene-based absorbents reported previously

**Samples**	**RL_{max} (dB)**	**EAB (GHz)**	**Refs.**
$Ti_3C_2T_x$	-40.3	3.66	[S1]
Amorphous carbon-$Ti_3C_2T_x$	-48.6	2.8	[S2]
Carbon nanosphere-$Ti_3C_2T_x$	-21.3	3.2	[S3]
Cellulose-$Ti_3C_2T_x$	-43.4	4.5	[S4]
Fe_3O_4-$Ti_3C_2T_x$	-53.4	1.5	[S5]
Ni-$Ti_3C_2T_x$	-49.9	2.1	[S6]
$Ni_{0.5}Zn_{0.5}Fe_2O_4$-$Ti_3C_2T_x$	-42.5	3.0	[S7]
PVB/$Ba_3Co_2Fe_{24}O_{41}$/$Ti_3C_2T_x$	-46.3	1.6	[S8]
ZnO-$Ti_3C_2T_x$	-26.3	1.4	[S9]
MXene-rGO/CoNi	-54.1	4.9	This work

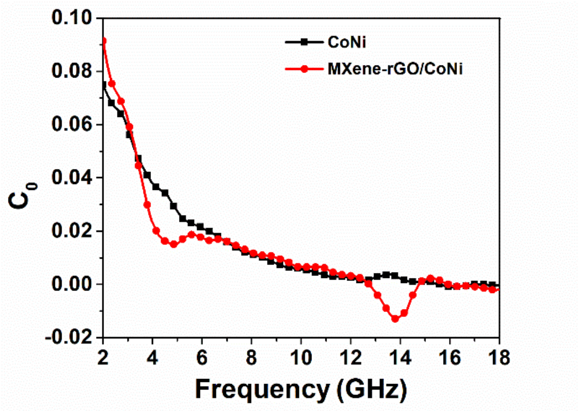

Fig. S10 The C_0 curves of rGO/CoNi and MXene-rGO/CoNi samples

Supplementary References

[S1] G. Cui, X. Zheng, X. Lv, Q. Jia, W. Xie et al., Synthesis and microwave absorption of $Ti_3C_2T_x$ MXene with diverse reactant concentration, reaction time, and reaction temperature. Ceram. Int. **45**(17), 23600-23610 (2019). https://doi.org/10.1016/j.ceramint.2019.08.071

[S2] M. Han, X. Yin, H. Wu, Z. Hou, C. Song et al., Ti_3C_2 MXenes with modified surface for high-performance electromagnetic absorption and shielding in the x-band. ACS Appl. Mater. Interfaces **8**(32), 21011-21019 (2016). https://doi.org/10.1021/acsami.6b06455

[S3] B. Dai, B. Zhao, X. Xie, T. Su, B. Fan et al., Novel two-dimensional $Ti_3C_2T_x$ MXenes/nano- carbon sphere hybrids for high-performance microwave absorption. J. Mater. Chem. C **6**(21), 5690-5697 (2018). https://doi.org/10.1039/c8tc01404c

[S4] Y. Jiang, X. Xie, Y. Chen, Y. Liu, R. Yang et al., Hierarchically structured cellulose aerogels with interconnected MXene networks and their enhanced microwave absorption properties. J. Mater. Chem. C **6**(32), 8679-8687 (2018). https://doi.org/10.1039/c8tc02900h

[S5] X. Zhang, H. Wang, R. Hu, C. Huang, W. Zhong et al., Novel solvothermal preparation and enhanced microwave absorption properties of $Ti_3C_2T_x$ MXene modified by in situ coated Fe_3O_4 nanoparticles. Appl. Surf. Sci. **484**, 383-391 (2019). https://doi.org/10.1016/j.apsusc.2019.03.264

[S6] L. Liang, G. Han, Y. Li, B. Zhao, B. Zhou et al., Promising $Ti_3C_2T_x$ MXene/Ni chain hybrid with excellent electromagnetic wave absorption and shielding capacity. ACS Appl. Mater. Interfaces **11**(28), 25399-25409 (2019). https://doi.org/10.1021/acsami.9b07294

[S7] Y. Li, X. Zhou, J. Wang, Q. Deng, M. Li et al., Facile preparation of in situ coated $Ti_3C_2T_x/Ni_{0.5}Zn_{0.5}Fe_2O_4$ composites and their electromagnetic performance. RSC Adv. **7**(40), 24698-24708 (2017). https://doi.org/10.1039/c7ra03402d

[S8] H. Yang, J. Dai, X. Liu, Y. Lin, J. Wang et al., Layered PAB/$Ba_3Co_2Fe_{24}O_{41}/Ti_3C_2T_x$ MXene composite: enhanced electromagnetic wave absorption properties with high impedance match in a wide frequency range. Mater. Chem. Phys. **200**, 179-186 (2017). https://doi.org/10.1016/j.matchemphys.2017.05.057

[S9] Y. Qian, H. Wei, J. Dong, Y. Du, X. Fang et al., Fabrication of urchin-like ZnO-MXene nanocomposites for high-performance electromagnetic absorption. Ceram. Int. **43**(14), 10757-10762 (2017). https://doi.org/10.1016/j.ceramint.2017.05.082
